# A retrospective cross-sectional study on district-based socioeconomic status and prostate cancer diagnosis

**DOI:** 10.1007/s00508-024-02449-8

**Published:** 2024-10-28

**Authors:** Ozan Yurdakul, Altug Tuncel, Melanie R. Hassler, Katharina Oberneder, David V. Gamez, Mesut Remzi

**Affiliations:** 1 Department of Urology, Medical University of Vienna, Währinger Gürtel 18–20, 1090 Vienna, Austria; 2 Department of Urology, Ankara State Hospital, University of Health Sciences School of Medicine, Ankara, Turkey; 3 Working Group on Laparoscopy and Robot-Assisted Surgery of the Austrian Society for Urology and Andrology (ÖGU), Vienna, Austria

**Keywords:** Prostate cancer, Cancer Screening, Prostate-specific antigen, Neighborhood disadvantage, Delivery of healthcare

## Abstract

**Introduction:**

Socioeconomic disparities have been linked to delayed prostate cancer diagnosis and poorer outcomes in various countries. This study aims to evaluate the socioeconomic disparities in prostate cancer diagnostics in Vienna, Austria, by examining initial prostate-specific antigen values and age at diagnosis across different districts and nationalities.

**Methods:**

This retrospective study included 1356 prostate cancer patients treated at the Medical University of Vienna between 2012 and 2022. Influence of residential districts and nationalities of the patients on the initial prostate-specific antigen (iPSA) value and on the age at diagnosis were analyzed. Patient data, including iPSA values, residential districts, and nationalities, were retrieved from the hospital’s internal documentation system. The information on average income of residential districts was obtained from the City of Vienna’s municipality data. Nationalities were grouped into EU and non-EU categories. Statistical analyses, including linear regression and t‑tests, were performed to examine the relationship between iPSA values, age at diagnosis, and socioeconomic variables. Linear regression was used to analyze the relationship between district income and both iPSA values and age at diagnosis.

**Results:**

The study found no significant differences in iPSA values and age at diagnosis between patients from higher income and lower income districts. Additionally, there were no significant differences among individual districts or between EU and non-EU nationals.

**Conclusion:**

The findings suggest that the Austrian healthcare system provides equitable access to prostate cancer diagnostics across different socioeconomic groups.

## Introduction

Prostate cancer (PCa) is the second most common malignancy among men globally [1]. In Austria PCa accounts for approximately one quarter of all new malignant neoplasms diagnosed in males and is responsible for nearly one eighth of all cancer fatalities in men [2]. Worse outcomes in PCa patients with lower socioeconomic status has mainly been attributed to delayed diagnosis, suboptimal diagnostic work-up and less invasive treatment [3]. In the diagnostics of PCa, the initial prostate-specific antigen (iPSA) value is an important prognostic factor as an important indicator for the state of the disease at the time of diagnosis [4].

The postal code has been suggested as a viable tool for measuring disparities in healthcare systems in urban areas [5]. The PCa level has been linked to delayed diagnosis and worse outcomes in poorer neighborhoods in many countries with different healthcare systems, such as USA, United Kingdom, Germany or Japan [6]. Similarly, in Austria variations in healthcare have been documented, such as districts at the lower end of the income scale exhibiting shorter life expectancies [7, 8]. Additionally, immigration and foreign nationality has been associated with adverse health outcomes in various countries, including the PCa setting [9].

The socioeconomic inequalities in PCa in Austria have not yet been explored. In the absence of data, this study aims to evaluate the socioeconomic disparities in the diagnostics of PCa in Austria. The differences in the iPSA values and age at diagnosis among the Viennese districts were compared. Moreover, the impact of foreign nationality on the iPSA values and age at diagnosis was evaluated.

## Methods

In this retrospective study all PCa patients residing in Vienna who received treatment at our center between 2012 and 2022 were included. The iPSA values, patients’ residential districts, and nationality were retrieved from our hospital’s internal documentation system. Hormonal treatment for PCa was not documented in any patient prior to the iPSA value. The average income values for each district were obtained from the municipality of the City of Vienna [10]. The residential districts and the nationality of the patients were obtained from the internal information system of our center. The variable nationality was classified into EU nationality and non-EU nationality. An immigration background was defined as being born outside the current EU borders and having previous or current non-EU citizenship.

### Statistical analysis

All statistical analyses were conducted using SPSS software, version 26 (IBM Corp., Armonk, NY. USA). A linear regression analysis was conducted to examine the relationship between iPSA values and average district income. In this analysis, the iPSA values served as the dependent variable, while the average income of the district was the independent variable. The income values of districts were treated as continuous variables. Additionally, a similar linear regression analysis was conducted with age at diagnosis as the dependent variable and average district income as the independent variable.

Based on data from the municipality of Vienna, the districts were categorized into two cohorts: one comprising patients from districts with an average income higher than the Vienna average, and the other from districts with a lower average income. To further detail the analysis, the richest four districts were also compared with the poorest four districts regarding the iPSA values and the age of diagnosis.

Statistical analysis of the iPSA values and age at diagnosis between the two groups were conducted using an independent t‑test. Additionally, to compare iPSA values and age at diagnosis among specific districts, a Kruskal-Wallis test followed by Dunn’s test with Bonferroni correction was performed. Furthermore, the differences in iPSA and age at diagnosis between patients with a non-EU nationality and those with EU nationality were analyzed using an independent t‑test. A *p*-value of less than 0.05 was considered as statistical significance.

## Results

### Patient demographics

The study included a total of 1356 PCa patients who received treatment at our department. The descriptive statistics, including residential districts, residential district group, nationality, and age at diagnosis, are presented in Table 1.

**Table 1:** Socioeconomic and diagnostic chararacteristics of the PCa patients in Vienna

Variable	Vienna	Lower income districts	Higher income districts
iPSA in ng/mL, median (SD)	8.7 (118.04)	8.8 (119.13)	8.6 (117.08)
Age of diagnosis (in years), median (SD)	68.6 (8.59)	68.3 (8.62)	69.1 (8.55)
Immigration background, *n* (%)	288/1356 (21.2%)	135/662 (20.4%)	153/694 (22.1%)
EU nationality, *n* (%)	1224/1356 (90.3%)	585/662 (88.4%)	639/694 (92.1%)

*SD* standard deviation

### iPSA values

**Fig. 1:**
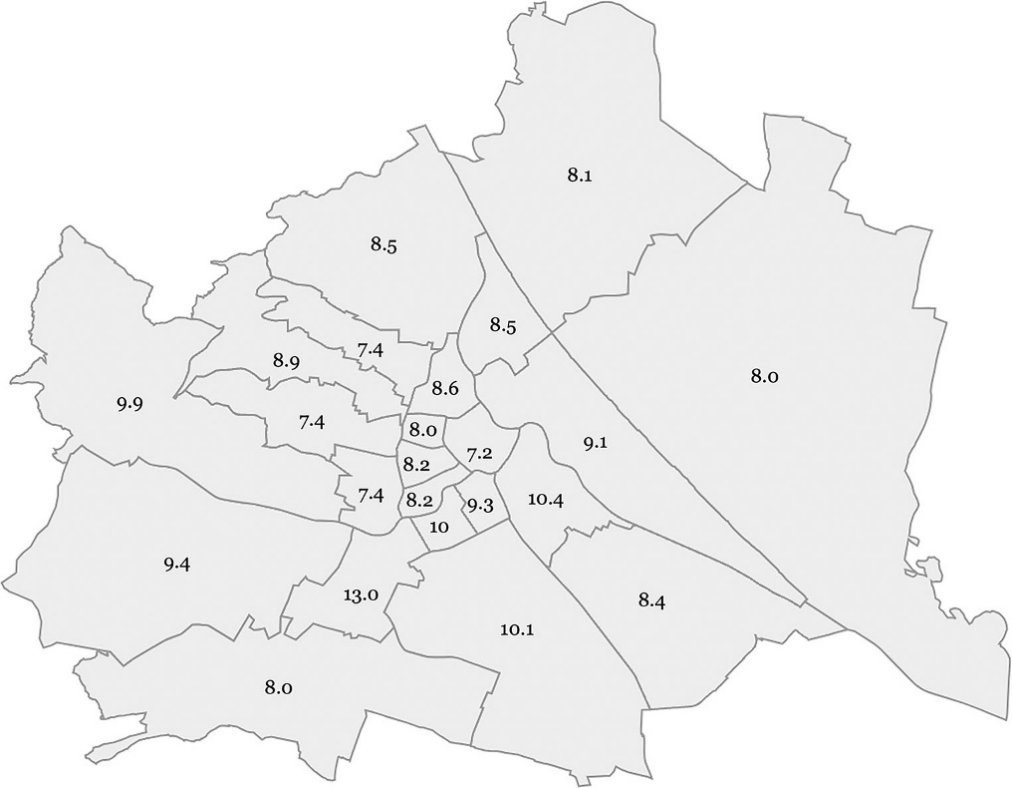
iPSA Map of Vienna. Map of Viennese districts with the median iPSA values in ng/mL

The regression analysis did not show a significant relationship between iPSA values and average district income (*p* = 0.61). The analysis revealed no significant difference in iPSA values between patients from higher income districts and those from lower income districts (*p* = 0.92). Additionally, there were no significant differences in iPSA values among the individual districts (*p* = 0.83). When comparing the iPSA values of the four highest income districts with those of the four lowest income districts, the statistical analysis also did not yield a significant difference (*p* = 0.785). The EU nationality did not impact the iPSA values of the patients (p *=* 0.998). Additionally, there was no statistically significant difference in iPSA values between specific nationalities (p = 0.445). The median iPSA value for each district is provided in Table 2. Moreover, an iPSA map of Vienna is provided in Fig. 1, and the distribution of nationalities with their respective median iPSA values is detailed in Table 3.

**Table 2:** Viennese districts with the median age of PCa diagnosis

District	Average net income per year* (in EUR)	Median iPSA (in ng/ml)	Median age at diagnosis (in years)
1010	37,872	7.20	68
1020	24,027	9.13	70
1030	27,133	10.45	68
1040	28,530	9.35	71
1050	22,533	10.00	69
1060	26,315	8.18	69
1070	27,612	8.15	68
1080	27,821	8.00	68
1090	27,028	8.60	69
1100	21,468	10.10	66
1110	22,967	8.45	68
1120	22,383	13.05	67
1130	32,592	9.39	70
1140	26,400	9.90	66
1150	20,122	7.40	68
1160	22,329	7.42	66
1170	23,634	8.00	69
1180	28,674	7.40	69
1190	30,141	8.50	72
1200	20,958	8.50	69
1210	24,843	8.14	68
1220	27,018	8.06	67
1230	28,075	8.00	68

The data depict the average net income of employed persons in Vienna, taken from the Viennese municipality [10]

**Table 3:** Distribution of nationalities

Nationality	*n* (%)	Median iPSA in ng/ml
Austrian	952 (70.21%)	8.5
German	68 (5.01%)	8.7
Polish	54 (3.98%)	9.4
Croatian	48 (3.54%)	8.9
Hungarian	43 (3.17%)	9.1
Romanian	22 (1.62%)	8.6
EU others	37 (2.73%)	9.2
Serbian	30 (2.21%)	8.8
Turkish	26 (1.92%)	9.0
Bosnian	16 (1.18%)	8.9
Non-EU others	60 (4.42%)	9.5

### Age at diagnosis

The linear regression analysis showed no significant relationship between average district income and the age of diagnosis (*p* = 0.165). There was no significant difference in the age at diagnosis between patients from higher income districts and those from lower income districts (*p* = 0.67). Additionally, no significant differences were found in the age at diagnosis among the specific districts (*p* = 0.75). When comparing the age of diagnosis of the four highest income districts with those of the four lowest income districts, the statistical analysis also did not yield a significant difference (*p* = 0.125). The data showed no significant differences in the age at diagnosis between EU nationals and non-EU nationals (*p* = 0.48). The median age at diagnosis for each district is provided in Table 2.

## Discussion

The influence of socioeconomic differences on the health outcomes has been demonstrated in different settings, and for different diseases [11]. Moreover, healthcare systems vary across different countries, meaning that the magnitude and character of health inequalities might differ around the globe. In Vienna, life expectancy varies markedly between residential districts. In the lowest income district (15th district), life expectancy is approximately 7 years shorter compared to the first district, which is the district with the highest income [7]. Also, disease-specific health discrepancies due to socioeconomic factors have also been reported in Vienna. For instance, ST-segment elevation myocardial infarction patients residing in lower income districts have been reported to present at a younger age [12]. Also, COVID-19 outcomes have been reported to be poorer among patients with disadvantaged socioeconomic status in Vienna [13]. It should be noted that the influence of environmental factors on the etiology of PCa is relatively low in comparison to cardiovascular diseases [14, 15].

Yet, also in PCa, socioeconomic factors have been associated with outcomes in multiple studies. For example, in Sweden, a nationwide population-based study suggested improved outcomes for the PC patients with higher income [16]. The study reported a significant impact of higher income on treatment outcomes, including a lower risk of positive margins after radical prostatectomy and reduced risk of PCa mortality; however, it did not assess the impact of income on possible differences at the time of diagnosis, such as age at diagnosis, or iPSA levels. A retrospective study of Freeman et al. in Chicago found the residential area to be a determinant of prostate-specific mortality [17]. But, also in this study, possible differences at the time of diagnosis were not assessed. A systematic review by Coughlin et al. assessing multiple retrospective studies found a substantial role of immigration background on stage at diagnosis and survival [18].

In the diagnostic setting of PCa, socially disadvantaged groups were demonstrated to have a lower incidence of PCa and more advanced stage at diagnosis, which can be explained by an inadequate diagnostic work-up of these populations [19]. Timely detection of clinically significant PCa is crucial for best possible treatment outcomes, making it important to identify socioeconomic barriers that hinder timely diagnosis. Testing of PSA is a well-established method that often triggers further diagnostics (most often biopsy) leading to PCa detection [20]. Socioeconomic disparities in PSA testing have been reported, with multiple studies showing that PSA testing is more prevalent in affluent residential areas [21, 22]. Additionally, immigration background has been linked to lower PSA testing frequencies [23]. Despite PSA being the standard marker for PCa and its correlation with disease state [20], to the best knowledge of the authors, this is the only study assessing the relationship between PSA levels and socioeconomic factors.

The absence of significant disparities in iPSA values and age at diagnosis among different socioeconomic groups in Vienna suggests that the Austrian healthcare system might be effective in providing equitable access to PCa diagnostics. These findings are interesting, especially when the abovementioned substantial differences in health outcomes in Vienna are taken into account. It should be noted that PSA screening for men over 45 years is covered by the mandatory health insurance in Austria, which covers 99% of the population [24]. In contrast, PSA screening in men is not reimbursed by the insurance in many European countries, such as Germany or France [25, 26]*.* Furthermore, the practical nature of the PSA screening as a blood test may account for the observed socioeconomic indifference in PCa diagnosis in Vienna.

The main limitations of this study were its retrospective and single-center nature. Considering that the Vienna General Hospital is a major referral center, potential selection bias may arise from differences between patients treated at the study center and those who were not. Using residential districts as proxies for socioeconomic status might miss individual nuances, and broad nationality classifications may overlook diversity within groups. It spans a decade during which healthcare policies have changed. Moreover, while current data suggest no significant disparities in PC diagnostics in Vienna, data regarding the differences in the outcomes of PCa are still absent. It should also be noted that staging of the patients was not evaluated in this study. More than half of the patients did not have documentation of imaging examinations. A substantial number of high-risk prostate cancer patients at our clinic (approximately 100 patients) underwent staging exclusively with prostate-specific membrane antigen positron emission computer tomography (PSMA-PET/CT), which further constrains the analysis due to its inherent differences from conventional staging methods. Consequently, due to the lack of high-quality data, the metastatic status at the time of diagnosis was not evaluated.

## Conclusion

This study found no significant differences in iPSA values and age at diagnosis of PCa among different socioeconomic districts in Vienna, nor between EU and non-EU nationals. These results suggest that the Austrian healthcare system provides equitable access to PCa diagnostics across socioeconomic groups; however, further research into treatment outcomes and broader socioeconomic factors is needed to ensure comprehensive healthcare equity.
